# Integration of ATAC-Seq and RNA-Seq Analysis to Identify Key Genes in the Longissimus Dorsi Muscle Development of the Tianzhu White Yak

**DOI:** 10.3390/ijms25010158

**Published:** 2023-12-21

**Authors:** Jingsheng Li, Zongchang Chen, Yanbin Bai, Yali Wei, Dashan Guo, Zhanxin Liu, Yanmei Niu, Bingang Shi, Xiaolan Zhang, Yuan Cai, Zhidong Zhao, Jiang Hu, Jiqing Wang, Xiu Liu, Shaobin Li, Fangfang Zhao

**Affiliations:** Gansu Key Laboratory of Herbivorous Animal Biotechnology, College of Animal Science and Technology, Gansu Agricultural University, Lanzhou 730070, China

**Keywords:** *Bos grunniens*, muscle, chromatin accessibility, ATAC-seq, RNA-seq

## Abstract

During the postnatal stages, skeletal muscle development undergoes a series of meticulously regulated alterations in gene expression. However, limited studies have employed chromatin accessibility to unravel the underlying molecular mechanisms governing muscle development in yak species. Therefore, we conducted an analysis of both gene expression levels and chromatin accessibility to comprehensively characterize the dynamic genome-wide chromatin accessibility during muscle growth and development in the Tianzhu white yak, thereby elucidating the features of accessible chromatin regions throughout this process. Initially, we compared the differences in chromatin accessibility between two groups and observed that calves exhibited higher levels of chromatin accessibility compared to adult cattle, particularly within ±2 kb of the transcription start site (TSS). In order to investigate the correlation between alterations in chromatin accessible regions and variations in gene expression levels, we employed a combination of ATAC-seq and RNA-seq techniques, leading to the identification of 18 central transcriptional factors (TFs) and 110 key genes with significant effects. Through further analysis, we successfully identified several TFs, including Sp1, YY1, MyoG, MEF2A and MEF2C, as well as a number of candidate genes (*ANKRD2*, *ANKRD1*, *BTG2* and *LMOD3*) which may be closely associated with muscle growth and development. Moreover, we constructed an interactive network program encompassing hub TFs and key genes related to muscle growth and development. This innovative approach provided valuable insights into the molecular mechanism underlying skeletal muscle development in the postnatal stages of Tianzhu white yaks while also establishing a solid theoretical foundation for future research on yak muscle development.

## 1. Introduction

The domestic yak (*Bos grunniens*) is the most famous livestock throughout much of the Asian highlands and the largest meat-producing mammal in the Qinghai–Tibet Plateau [1]. As a pivotal livestock species in this region, domestic yaks provide essential resources such as meat, milk, transportation and fuel [2,3,4,5]. However, the production performance of yak milk and yak meat is comparatively lower when compared to that of cattle-yak and ordinary cattle [6,7]. Therefore, enhancing the growth rate and meat production performance of yak has been a primary focus in yak breeding research.

Skeletal muscle is the main meat-producing tissue, which constitutes 40% of the total body weight in adult bovines, and is directly related to the meat producing capacity of them [8]. The development of skeletal muscle is a complex and intricately orchestrated biological process regulated by a series of specific signaling pathways and transcription factors (TFs), encompassing cell proliferation, differentiation, fusion, migration and apoptosis [9,10,11]. In addition to the substantial involvement of diverse myogenic regulators in skeletal muscle development, epigenetic modifications play a crucial role in facilitating muscle growth [8,12,13]. For instance, m6A modification intricately and negatively governs the expression levels of pivotal genes implicated in the growth and development of skeletal muscle in yaks [14]. The regulation of muscle growth and development is achieved through the methylation of gene promoters, which in turn controls the expression of genes associated with muscle function [15,16]. However, an investigation of chromatin accessibility during various stages of postnatal muscle growth and development in yaks has not been undertaken to elucidate alterations in gene expression [17,18,19,20].

Eukaryotic DNA is not bare and its genomes undergo extensive compaction within chromatin, which is known as closed chromatin [21]. When transcription of the DNA within a cell commences, the chromatin region encompassing transcriptional regulatory elements assumes an open conformation, rendering the DNA exposed and giving rise to open chromatin regions (OCRs). This state of openness facilitates the binding of transcription factors (TFs) to the DNA within OCRs, thereby exerting control over the process of transcription [17,22,23,24]. Binding sites for TFs and chromatin regulators are primarily located in regions of the genome that are open or accessible. Assay for Transposase-Accessible Chromatin sequencing (ATAC-seq), currently considered the preferred method for studying epigenetics, enables us to create a sensitive profile of chromatin accessibility by directly transposing native chromatin within the intact nucleus and analyzing the binding patterns of various TFs [25,26,27]. Recently, several researchers have conducted investigations into skeletal muscle development in mice, pigs and cattle through the utilization of RNA sequencing (RNA-seq) and chromatin accessibility analysis [18,28,29,30]. For instance, the integration of RNA-seq and ATAC-seq identified 22 candidate hub genes potentially targeted by MEF2C during bovine skeletal muscle development [19]. Moreover, previous studies have delineated dynamic alterations in chromatin accessibility and gene expression, as well as identified numerous instances of transcription factor binding events during the proliferation and differentiation of bovine myoblasts in vitro, encompassing ATF3, MyoG, AP-1, ZBTB18, Myf5 and HLH-1 [18]. However, there has been limited investigations into the alterations in chromatin accessibility and gene expression during yak skeletal muscle development.

In this study, we investigated the epigenetic variations in the longissimus dorsi muscle of yak at two postnatal time points to gain a deeper understanding of the underlying mechanisms involved in skeletal muscle development. By employing ATAC-seq and RNA-seq techniques, we further established a correlation between chromatin accessibility and gene expression. These findings offer novel insights into the epigenetic regulatory mechanisms that govern skeletal muscle development, thereby providing valuable references for genetic breeding strategies in yaks.

## 2. Results

### 2.1. ATAC-Seq Quality Control of the White Yak Longissimus Dorsi Muscle

**Figure 1:**
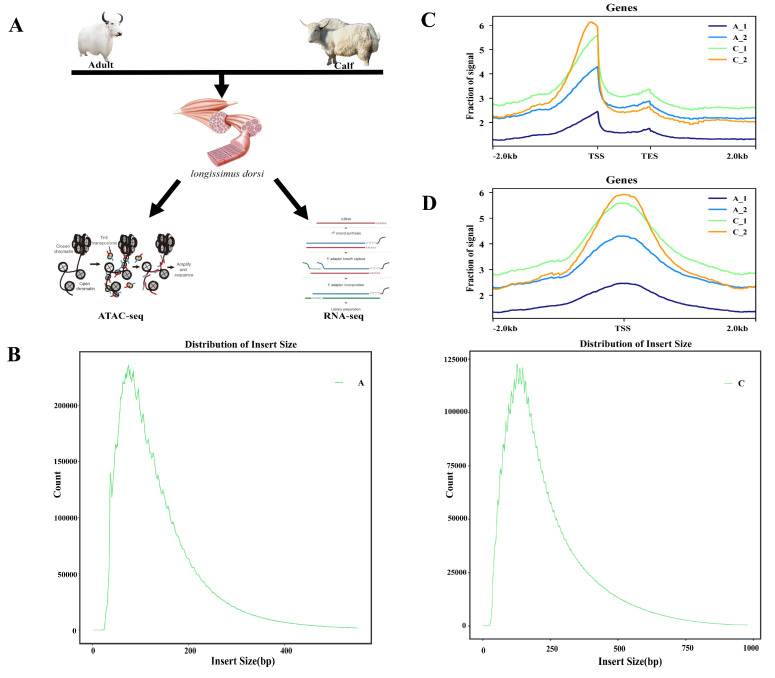
Phenotype and ATAC-seq quality control. (**A**) Overview of the experiment. Longissimus dorsi tissue from adult and calf yaks were collected for ATAC-seq and RNA-seq. In the ATAC-seq schematic the ellipse with red and blue fragments represents the Tn5 transposase, the circle with the black fragment represents the chromatin, and the circle with red and blue represent the cleaved chromosome fragments. (**B**) Fragment length distribution map. (**C**,**D**) Distribution of mapped reads across gene bodies and peaks.

To elucidate the chromatin accessible regions across the whole genome involved in muscle growth and development, we characterized the differential chromatin accessibility between the calf yaks (Calf group, C) and the adult yaks (Adult group, A) by using ATAC-seq (Figure 1A) [25,31]. We obtained a total of 430,228,222 raw reads, of which 427,139,652 clean reads were uniquely mapped to the reference genome after filtration (Table 1). We evaluated the quality of the libraries by analyzing the lengths of inserted fragments and peak signal distributions. Fragment size analysis showed that the majority of fragments were <250 bp in length, including one mononucleosome fragment and one nucleosome-free fragment, indicating that all libraries are available for subsequent experiments (Figure 1B and Figure S1). DeepTools software (version 2.5.4) was used to calculate the distribution of reads across the gene bodies and peaks. The majority of the accessible regions were located within ±2 kb of the transcription start site (TSS), suggesting that open chromatin regions were essential for gene transcriptional regulation (Figure 1C,D). These results demonstrated the high quality of the sequencing data.

### 2.2. Chromatin Accessibility in the Longissimus Dorsi Muscle of the White Yak

**Figure 2:**
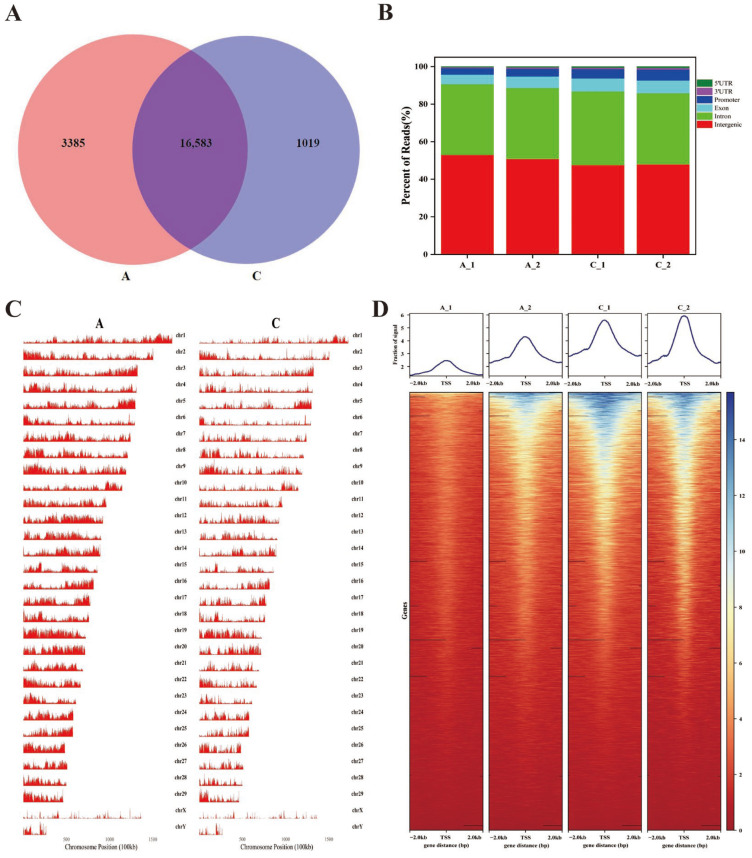
Distribution of peaks. (**A**)Venn diagram showing the peak overlap between A and C groups. (**B**) Chromosomal distribution of all peaks. (**C**) Genomic distribution of the peaks in each sample. Genomic functional regions include promoter, intergenic, exon, intron, 5′UTR and 3′UTR. (**D**) A heatmap of the peak signals across the gene body of library; ±2.0 represents upstream and downstream of the TSS.

We identified 3385 and 1019 specific accessible chromatin peaks, and 16,583 common peaks in A and C groups (Figure 2A, Table S1). The distribution of chromosomal peaks in the yak genome is illustrated in Figure 2B. The chromatin open region maps at the chromosome level in the whole genome showed that most of the regions on each chromosome, including the X chromosome and the Y chromosome, were covered with peaks. To annotate the genomic distribution of open chromatin peaks, they were assigned to genome-wide functional regions, including 5’ untranslated regions (UTR), intergenic, promoters, introns, exons and 3′UTR. Most peaks were mapped in promoter regions, intergenic, exon and intron regions. We have discovered that the peaks in the promoter regions of C accounted for 5.37%, while those in A accounted for 3.72% of the total area (Figure 2C, Table S2). The heatmap indicated an increased enrichment in the readings within regions ±2 kb of the TSS and termination end sites (TES) in the genome. The heatmap showed that the ATAC-seq signals for calf yaks were stronger than for adult yaks, which suggested that these strong signal peaks may be pivotal sites in the regulation of muscle development (Figure 2D).

**Figure 3:**
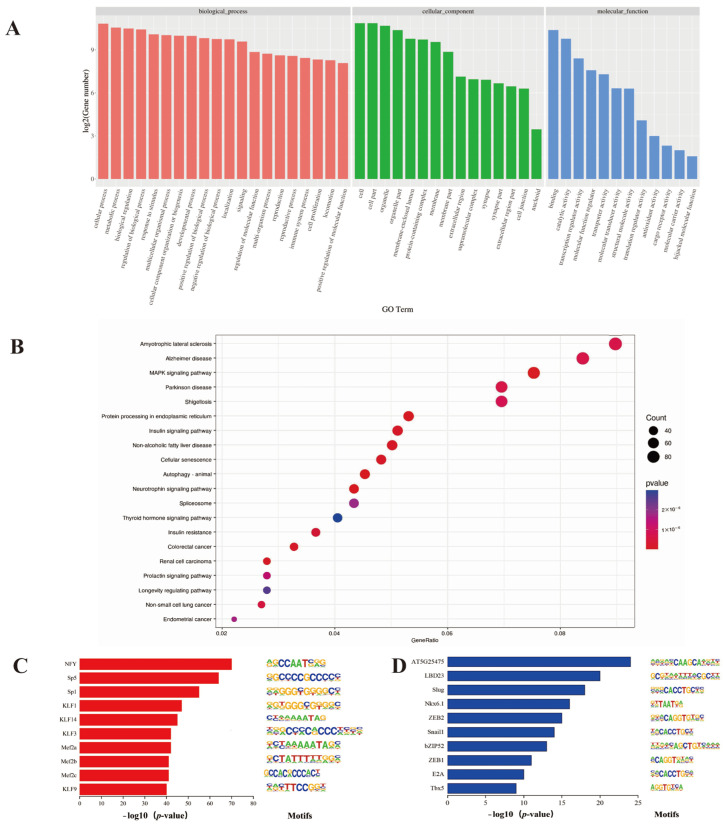
GO and KEGG pathway enrichment analysis and motifs analysis of genes associated with differential chromatin accessibility. (**A**) GO enrichment analysis of genes corresponding to differential peaks. (**B**) KEGG pathway enrichment analysis of genes corresponding to differential peaks. (**C**) Enriched transcription factor binding motifs by increased peaks (*p* < 0.01). (**D**) Enriched transcription factor binding motifs by decreased peaks (*p* < 0.01). Green A stands for adenine, red T for thymine, blue C for cytosine and yellow G for guanine.

Annotating each peak revealed that the different peaks in the C group correspond to 2024 upregulated genes and 18 downregulated genes, and a total of 2224 genes were obtained compared with the A group (Table S3). To explore the potential functions of these genes, we conducted GO and KEGG enrichment analysis. We found that the biological processes in GO analysis were mainly related to cellular processes, developmental processes and cell proliferation, whereas molecular functions were mainly enriched in catalytic activity and transcription regulator activity (Figure 3A, Table S4). In addition, the KEGG enrichment analysis revealed that differentially expressed genes (DEGs) were enriched in pathways associated with body growth and development, such as the MAPK signaling pathway, the insulin signaling pathway and the thyroid hormone signaling pathway (Figure 3B, Table S5). The growth rate of calf yaks is faster than that of adult yaks. Based on this, Homer software was used to analyze transcription factor binding motifs on different peaks in the yaks and compare them with mammalian transcription factors databases. As a result of increased peaks, the top 10 significantly enriched transcription factor binding motifs were identified as NFY, Sp5, SP1, KLF1, KLF14 and the MEF2 family (Figure 3C, Table S6). In contrast, ZEB2, ZEB1 and E2A were enriched by decreased peaks (Figure 3D, Table S7).

### 2.3. RNA-Seq Data from Longissimus Dorsi Muscle of the White Yak

**Figure 4:**
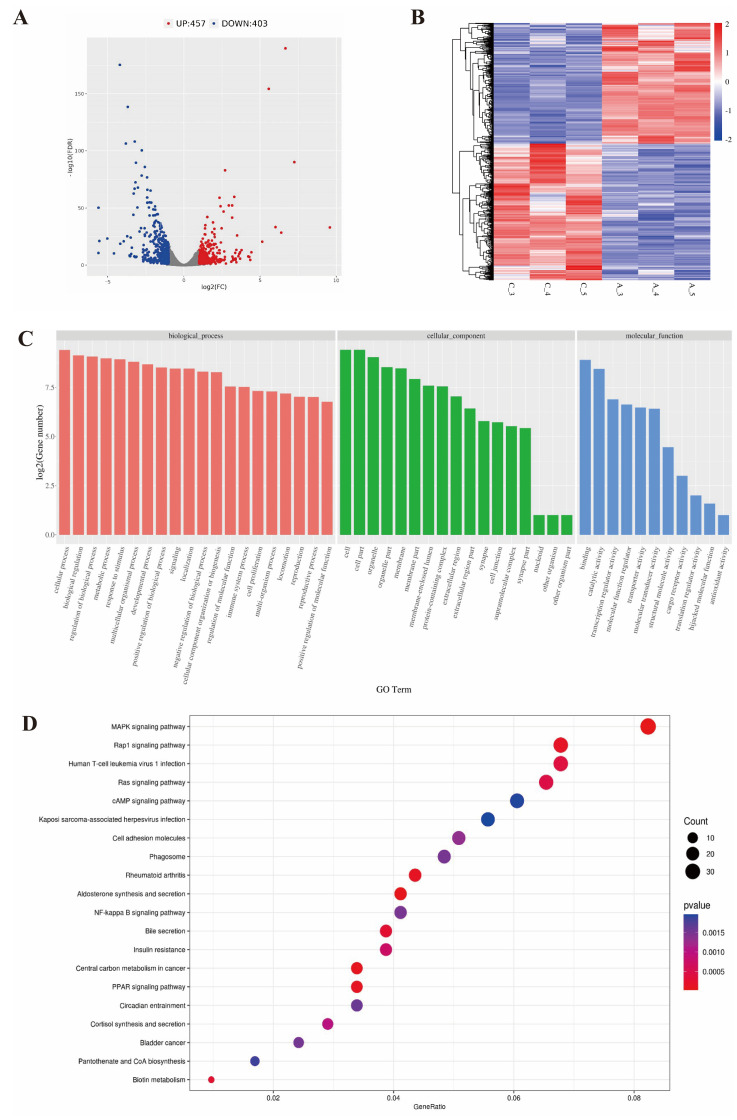
An analysis of DEGs by RNA-seq. (**A**) A volcano plot for DEGs. (**B**) Heatmap of the differentially expressed genes. (**C**) GO enrichment analysis of differentially expressed genes. (**D**) Bubble chart of KEGG pathway enrichment analysis of differentially expressed genes.

To determine gene expression patterns in yaks at different time periods, we selected three samples from the calf group and three samples from the adult group for high-throughput mRNA sequencing. A total of 489,332,176 clean reads were obtained from six transcriptome sequencing libraries (Table 2). To identify the vital functional genes, DEGs were filtered based on the requirements of |log_2_ (Fold Change)| ≥ 1 and FDR < 0.05. The volcano map showed 860 differential genes, including 457 upregulated genes and 403 downregulated genes (Figure 4A, Table S8). We determined the clustering pattern of DEmRNAs. The same group of DEGs was clustered together in the heatmap, indicating the accuracy and reliability of the samples (Figure 4B). The potential function of these DEGs was investigated by employing GO and KEGG pathway enrichment analysis. The GO enrichment results showed that these DEGs were mainly enriched in cell proliferation, the regulation of biological processes and transcription regulator activity (Figure 4C, Table S9). Several KEGG pathways related to energy metabolism and cell signaling transduction were significantly enriched, such as the MAPK signaling pathway, cAMP signaling pathway, Rap1 signaling pathway, PPAR signaling pathway and Pantothenate and CoA biosynthesis. These results suggested that DEGs may play a role in the maintenance of muscle balance and the growth of muscle (Figure 4D, Table S10).

### 2.4. Combined Analysis of ATAC-Seq and RNA-Seq

**Figure 5:**
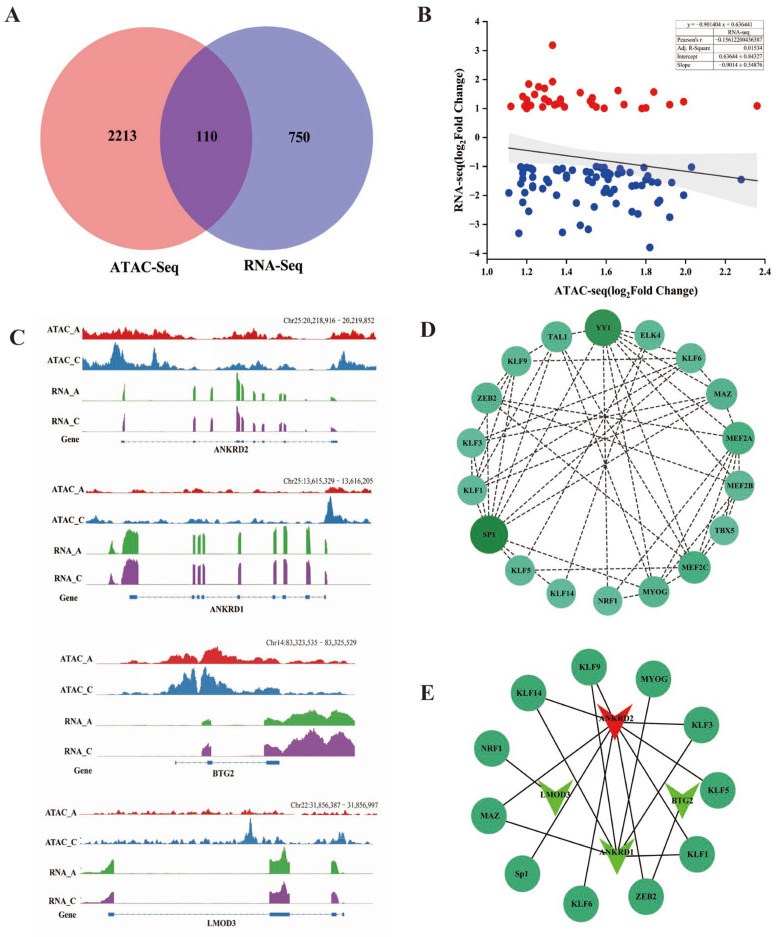
Integration of RNA-Seq and ATAC-Seq. (**A**) Venn plot of ATAC-seq and RNA-seq;110 common genes were found. (**B**) Correlation of significantly differentially accessible gene (ATAC-seq) and gene expression (RNA-seq). Red indicates up-regulated genes, blue indicates down-regulated genes. (**C**) IGV snapshot for ATAC-seq and RNA-seq signal for the *ANKRD2*, *ANKRD1*, *BTG2* and *LMOD3* gene. (**D**) The interaction network between transcription factors. The size of the nodes and the shade of the color represent the importance of the interaction between TFs (*p* < 0.01). (**E**) The interaction network between hub TFs and key genes. The dark green circle type represents TFs. The inverted triangles type represents genes, in which red represents upregulation, and the green represents downregulation.

In order to ascertain whether alterations in chromatin accessible regions correlated with variations in gene expression levels, we combined ATAC-seq and RNA-seq data. A total of 110 DEGs were identified, including 76 downregulated genes and 34 upregulated genes (Figure 5A, Table S11). We analyzed the correlation between the chromatin openness and expression levels of these 110 key genes, and found that the expression levels of DEGs were negatively correlated with differential ATAC-seq signaling (Pearson’s R = −0.15612, *p* < 0.001; Figure 5B, Table S12). To visually show the relationship between chromatin accessibility and gene expression, IGV was used to demonstrate the ATAC-seq and RNA-seq signaling of the genes *ANKRD2*, *ANKRD1*, *LMOD3* and *BTG2*, which have been deemed to be related to the development of muscle [31,32,33,34,35]. The ATAC-seq signals were significantly higher in the promoter region in the vicinity of the TSS than in the other regions of the genes. In the A group, both the chromatin accessibility and transcription levels of ANKRD2 were lower compared to the C group. In contrast, *ANKRD1*, *LMOD3* and *BTG2* had high chromatin accessibility but low transcription levels (Figure 5C). TFs can bind promoters with other regulatory proteins and cooperate with RNA polymerase II to start gene transcription and regulate gene expression. Based on this, all upregulated and downregulated peaks sequences were predicted through the Homer software motif. We screened the top 30 upregulated and down-regulated motifs that were significantly enriched in genes, and constructed a regulatory map of the transcription factor binding site network in yak muscle tissue by comparing the yak reference genome with the STRING database. We found that the transcription factor (TFs) Sp1, YY1, MYOG, MEF2A and MEF2C were highly correlated with other TFs in the network, suggesting their potential significance in the regulation of yak muscle development (Figure 5D, Table S13). Based on the above analysis results, we constructed an interaction network between four key genes and 18 transcription factor binding sites (Figure 5E, Table S14). A total of 11 transcription factors interact with the promoter regions of *ANKRD2*, *ANKRD1*, *LMOD3* and *BTG2*.

### 2.5. Validation of the Results by RT-qPCR

**Figure 6:**
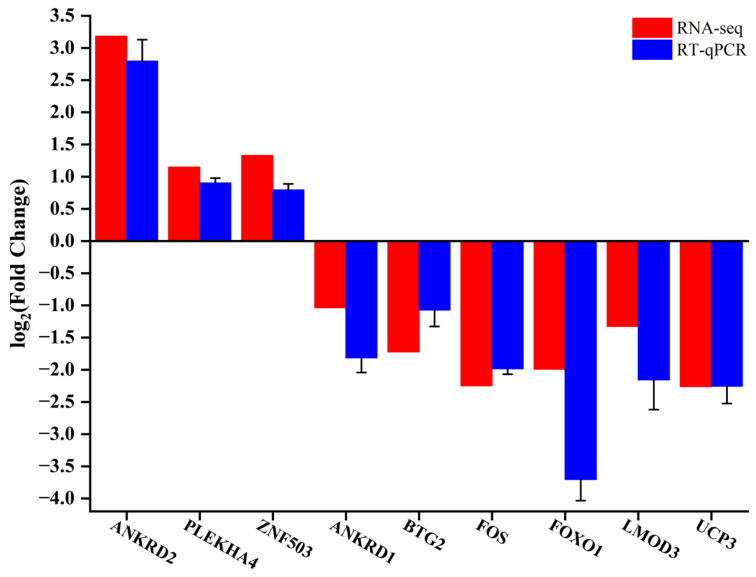
Verification of RNA-seq data by RT-qPCR. Histogram of RNA-seq and RT-qPCR expression levels. X-axis represents 9 key genes, and Y-axis represents the expression levels of genes from RNA-seq and RT-qPCR.

In order to confirm the precision of the RNA-seq data, we randomly selected nine genes (*ANKRD2*, *PLEKHA4*, *ZNF503*, *ANKRD1*, *BTG2*, *FOS*, *FOXO1*, *LMOD3* and *UCP3*) from DEGs for RT-qPCR. The results indicated that the RT-qPCR expression pattern of these genes corresponded with that of RNA-seq, suggesting the reliability of the DEGs identified by RNA-seq in this study (Figure 6).

## 3. Discussion

The yak, a rare breed of plateau cattle worldwide, has become the primary source of sustenance and livelihood for local herdsmen [36]. Given their slow growth rate and the low meat yield from yaks, there is a pressing need to expedite their growth in order to enhance meat production. In recent years, several research teams have investigated the transcriptional level mechanism of yak muscle development, providing fundamental insights for further exploration [6,14,37]. Building upon previous studies, we used the ATAC-seq and RNA-seq analysis methods for the first time to effectively identify the key factors influencing muscle development from a chromatin accessibility perspective, and explored the potential molecular mechanisms underlying the variations in skeletal muscle growth and development in the Tianzhu white yak.

In our study, we selected 12-month-old yaks exhibiting rapid growth and 4-year-old adult yaks as ideal models for investigating the postnatal skeletal muscle growth rate in yaks [37,38,39]. We identified a total of 19,968 upregulated and 17,602 downregulated accessible chromatin peaks between these two groups, which corresponded to 2024 upregulated genes and only 18 downregulated genes. We observed that the C group exhibited a higher ATAC signal value within ±2 kb of the TSS in compared to the A group. Previous studies on early embryonic development in cattle have demonstrated a significant increase in ATAC signal values at TSS sites during the embryonic genome activation [17,40]. In addition, it has been found that yaks grow slowly from 6 months to 12 months of age after birth, after which they enter a period of rapid growth [38,39]. Hence, we hypothesized that the substantial enhancement in chromatin accessibility at TSS region during muscle growth of 12-month-old white yaks may be associated with genome activation processes. Furthermore, we noted a similar distribution pattern of peaks in the gene 5′UTR and promoter regions as depicted by the line graph. ATAC-seq peaks exhibited significant enrichment within promoter region, aligning with previously reported chromatin accessibility features observed in the longissimus muscle of Duroc and Luchuan pigs [20,41]. These findings suggested a pivotal role for promoter, enhancer and cis-regulatory elements in the regulation of muscle development. Furthermore, another study has revealed the higher prevalence of muscle affinity peaks in the exon and promoter regions while identifying differentially expressed genes (DEGs) that are specifically expressed in the skeletal muscle of cattle using the RNA-seq and ATAC-seq techniques [19]. Our study yielded similar findings, enhancing our comprehension of chromatin accessibility and gene dynamic transcriptional regulation during skeletal muscle development.

The GO enrichment analysis of genes associated with peaks revealed that differential chromatin accessibility was primarily associated with cellular development and cell proliferation processes, while molecular functions were predominantly enriched in catalytic activity and transcription regulator activity. Furthermore, the KEGG pathway enrichment analysis demonstrated a significant correlation between differential chromatin accessibility and muscle growth and development, such as the MAPK signaling pathway, the insulin signaling pathway and the thyroid hormone signaling pathway. These findings further support the notion that skeletal muscle growth and development involves a complex transcriptional regulatory process controlled by multiple tightly regulated changes in gene expression. Interestingly, the enrichment result obtained in this study exhibits a remarkable similarity to the differential gene enrichment findings observed in RNA-seq analysis. By integrating the analysis of chromatin accessibility and transcriptome gene enrichment, we have successfully elucidated several crucial pathways implicated in the regulation of muscle development. Among these pathways, the MAPK signaling pathway, Rap1 signaling pathway and PPAR signaling pathway are recognized as the classical regulators of muscle development. The p38 MAPK signaling pathway plays a pivotal role in regulating muscle satellite cell proliferation and promotes skeletal muscle regeneration and differentiation by mediating the expression of myogenesis-related genes and epigenetic regulators [42,43]. Rap1, a widely distributed protein that belongs to Ras family, plays crucial roles in cellular proliferation and migration by serving as an upstream regulator that activates diverse signal transduction pathways [44,45]. Peroxisome proliferator-activated receptors (PPARs) play a crucial role in development and energy metabolism, as well as in the regulation of satellite cells proliferation, skeletal muscle regeneration, and diversification of muscle fiber types [46]. Furthermore, we have identified that certain hormones and coenzyme biosynthesis processes, such as insulin, thyroid hormone and pantothenate and CoA biosynthesis, also exert regulatory control over muscle development. Thyroid hormones are implicated in muscle contraction, metabolic activities and the growth and repair of skeletal muscle tissue [47]. The regulation of intracellular triiodothyronine levels exerts a direct influence on myogenesis by modulating the growth and differentiation of precursor cells [48]. Insufficient intramuscular T3 levels below the optimal threshold can lead to a decrease in energy metabolism in the muscles as well as reduced contraction and relaxation rates [49]. This biological process may involve the interplay among various bioactive substances, including the thyroid hormone and pantothenate and CoA biosynthesis [50,51].

We have integrated ATAC-seq data with RNA-seq data, revealing a significant correlation between chromatin accessibility and the expression levels of 110 genes. Furthermore, our combined analysis demonstrated the negative associations between differential gene expression and ATAC-seq signaling. This may be attributed to the actions of activating transcription factors, transcriptional repressors, DNA methylation or other epigenetic modifications in regulating gene expression [13,52,53,54,55]. We focused on four crucial genes located in differentially accessible chromatin regions (DARs) within gene promoters and exons of *ANKRD2*, *ANKRD1*, *BTG2* and *LMOD3*, which have been previously associated with muscle development [32,33,34,35]. Through gene expression analysis, we identified the *ANKRD2* gene as the top hub gene. In myoblasts, it predominantly localizes in the nucleus and upon differentiation, and translocates from the nucleus to the cytoplasm. This dynamic localization suggests its involvement in the coordination myoblast differentiation and proliferation [56]. Previous studies have also demonstrated the localization or close proximity of *ANKRD2* to the identified QTL region influencing meat quality and carcass traits, suggesting its potential as a candidate gene with significant influence on porcine growth and carcass traits [57]. These findings highlight the crucial role of the *ANKRD2* gene in promoting muscle development in Tianzhu white yaks.

Open chromatin regions (OCRs) serve as binding sites for transcription factors, initiating target gene transcription and playing a crucial role in myoblast proliferation and differentiation. In this study, we investigated the variability of transcription factors within OCRs at two time points. Our findings revealed that special TF motifs, including Sp1, YY1, MYOG, MEF2A and MEF2C, were enriched in the differentially accessible chromatin region. The TFs in this group are directly associated with the proliferation and differentiation of skeletal muscle cells. Sp1, a ubiquitously expressed mammalian transcription factor, plays a pivotal role in maintaining genome integrity through diverse mechanisms [58,59]. Recent research has revealed that Sp1 is essential for the assembly of mitotic chromosomes, thereby facilitating cell proliferation [60]. In vivo studies on YY1 knockout mice derived from skeletal muscle satellite cells have demonstrated that inducible deletion of YY1 significantly impedes the process of muscle repair caused by acute damage and exacerbates the dystrophic phenotype resulting from chronic injury [61]. MEF2A, a member of the MEF2 gene family which includes MEF2B, MEF2C and MEF2D [62], is a bHLH domain transcription factor highly expressed in the brain, skeletal muscle and heart muscle that plays an important role in regulating proliferation and differentiation of muscle cells [63]. MyoG, a member of the myogenic regulatory factors (MRFs) family, plays an indispensable role in individual growth, development and muscle formation by regulating the expression of creatine kinase, troponin and the troponin gene [64]. MEF2A and MyoG TFs can bind to the core promoter region of the bovine *LATS2* gene influencing its transcriptional activity and controlling animal muscle growth and development [65]. Based on these aforementioned findings, we hypothesize that Sp1, MyoG, MEF2A and MEF2C may serve as pivotal transcription factors (TFs) involved in regulating muscle growth in the Tianzhu white yaks. As initiators of transcriptional processes, TFs recognize specific sequences (motifs) within their target genes’ regions to regulate gene expression [66]. Therefore, we constructed networks of hub transcription factors (TFs) and crucial target gene interaction. We observed interactions between TFs and multiple genes in the regulatory networks. In future studies, a series of experiments should be conducted to further explore the regulatory effects of candidate TFs on target genes and the biological processes involved in skeletal muscle development of the Tianzhu white yak. Additionally, it is essential to analyze the underlying regulatory mechanism and elucidate the mode of action of these TFs and genes in muscle growth and development.

## 4. Materials and Methods

### 4.1. Collection of Samples

The yaks in the experiment were reared under identical breeding conditions. Five 4-year-old yaks and five 12-month-old white yaks were selected for slaughter to obtain the longissimus dorsi muscle samples used in this study. The samples were then divided into 0.5 cm^3^ portions, immediately frozen in liquid nitrogen and stored at −80 °C for further experiments. Here, two adult and two calf samples were sequenced for ATAC-seq and three adult and three calf samples were sequenced for RNA-seq.

### 4.2. ATAC-Seq

The ATAC-seq experiments were performed according to previously described methods [25,27,67]. After pulverizing the frozen tissue, approximately 3 g of longissimus dorsi muscle were isolated and immediately homogenized in 2 mL of pre-cooled lysis buffer. The resulting mixture was then ground and layered onto the surface of 2 mL of concentrated sucrose buffer in a 10 mL Falcon tube. Subsequently, the nuclei were centrifuged at 2200× *g* at 4 °C for 15 min and the resulting pellets were resuspended in 500 μL of pre-chilled lysis buffer.

The crude nuclei were re-suspended in the transposition mix (25 μL of reaction buffer, 2.5 μL of Nextera Tn5 Transposase and 22.5 μL of Nuclease free water) and incubated at 37 °C for 30 min. After transposition, DNA was purified using a QIAGEN MinElute PCR Purification Kit. It was worth noting that throughout these procedures, samples were consistently kept on ice.

NEBNext^®^ High-Fidelity 2 × PCR Master Mix was employed for DNA amplification, followed by purification of the amplified libraries using QIAGEN MinElute PCR Purification Kit (Hilden, Germany) according to the manufacturer’s instructions. The library was subsequently washed with 20 μL of elution buffer containing 10 mM Tris buffer at pH 8. Quality assessment of purified libraries was performed using Agilent 2100 (Santa Clara, CA, USA) and Thermofisher Qubit 4 Fluorometer (Waltham, MA, USA). Multiplex libraries were sequenced on the Illumina NovaSeq^TM^ 6000 next-generation sequencing platform (San Diego, CA, USA).

The adapter sequences and the low-quality reads were removed from the raw data using Trimomatic (version 0.36) and FASTQC (version 0.11.5), respectively. Subsequently, the clean reads were aligned to the reference genome of *Bos grunniens* (LU_Bosgru_v3.0) using HISAT2 (version2.0.1-beta) [68]. Peaks were identified using MACS2 software (version 2.1.0) default parameters and their identification was performed utilizing DeepTools (version 2.5.4). If a peak midpoint fell within the range of ± 5000 bp around the transcription start site (TSS) of a gene, it was assigned to that specific gene.

The heat maps and average map of the ATAC-seq data were generated using DeepTools (version 2.5.4). The distribution of peaks on gene functional elements was mapped using the ChIPseeker R package (Version 1.30.3). Peak differences between groups were analyzed using the DiffBind package (version 1.16.3), with criteria set at FDR < 0.05 and Fold > 0. Motif analysis was performed using Homer (version 3) and MEME-FIMO (version 4.11.2). Two biological replicates were utilized for ATAC-seq.

### 4.3. RNA-Seq

We employed the conventional Trizol method [69], in conjunction with the RNAprep Pure Tissue Kit DP431 (TIANGEN, Beijing, China), to performed total RNA extraction from yak longissimus dorsi muscle. Subsequently, all RNA samples were assessed for both integrity and concentration using 1.2% agarose and Thermofisher Qubit 4 Fluorometer respectively. The library construction process encompassed several steps including polyA-selected RNA extraction, RNA fragmenting and reverse transcription utilizing random hexamer primers. These procedures were executed employing the VAHTS mRNA-seq V3 Library Prep Kit for Illumina platform. Finaly, sequencing of libraries was performed on an Illumina Novaseq™ 6000 instrument generating paired-end reads of 150 nt length. Adapters and low-quality reads were subjected to filtration using Cutadapter (version 1.11). The resulting clean reads were subsequently aligned to the *Bos grunniens* reference genome using HISAT2. Transcript abundance was estimated and expression values were normalized using FeatureCounts (version 1.6.0) to obtain FPKM values [70]. Significantly differentially expressed genes were identified using edgeR (Version 3.36.0) with a filter threshold of FDR < 0.05 and |log_2_FoldChange| > 1. Three biological replicates were used for RNA-seq.

### 4.4. Integration Analysis of ATAC-Seq and RNA-Seq

The reads associated with each ATAC-seq peak were converted to RPKM (reads per kilobase per million mapped reads). Subsequently, the gene expression levels (FPKM) were determined for each group using consistent methodologies. We employed the same approch to partition the FPKM mRNA values for all genes and ascertain the RPKM values at distinct gene positions. The IGV software (version 2.12.2) was utilized to visualize the genome browser view of the merged datasets comprising ATAC-seq and RNA-seq data. Motif regulatory networks were constructed by utilizing the STRING database (Version 12.0) and displayed in Cytoscape (Version 3.10.1).

### 4.5. Gene Functional Annotation

The GO and KEGG enrichment analysis was conducted using the ClusterProfiler R package (Version 4.2.2) and the hypergeometric distribution approach with a significance level of *p* < 0.05.

### 4.6. RT-qPCR

We randomly selected 9 key genes and analyzed their expression using RT-qPCR. The forward (F) and reverse (R) primers for the genes used in this study were shown in Table 3. We utilized the HiScript^®^ III RT SuperMix for qPCR (+gDNA wiper) (Vazyme, Nanjing, China) to generate cDNA. Genomic DNA remaining was eliminated at 42 °C for 2 min, followed by a reverse transcription reaction at 37 °C for 15 min and 85 °C for 5 s. The PerfectStart^®^ Green qPCR SuperMix (Transgen, Beijing, China) was employed for real-time quantitative PCR (RT-qPCR), with the internal reference gene *GAPDH* utilized for data standardization. Each sample analyzed using RT-qPCR underwent a minimum of three biological repetitions. The 2^−ΔΔCt^ method was used to calculate and analyze the relative mRNA expression.

### 4.7. Statistical Analysis

The data were presented as the means ± standard deviation (SD) obtained at least three biological repeat samples. To compare the significance of mean values, paired t-tests (between two groups) or ANOVA (among multiple groups) were conducted using SPSS 26.0. The differences were regarded as very significant or significant at *p* < 0.01 or *p* < 0.05, respectively. The analysis of the results and generation of the images were carried out using the Origin 2023b software.

## 5. Conclusions

This study presents a comprehensive map of chromatin accessibility during muscle development in the Tianzhu white yaks, highlighting key genes and transcription factors (TFs) involved in this process. By comparing the differences in chromatin accessibility and gene expression between calves and adult white yaks, specifically in the longissimus dorsi, we constructed an interactive network program that integrates hub TFs and key genes associated with yak muscle development, providing a novel approach for understanding the postnatal stages of yak muscle development and establishing a theoretical foundation for future research.

## Figures and Tables

**Table 1:** Quality control for the ATAC-seq data.

Sample	Raw Reads	Raw Bases	Clean Reads	Clean Bases	Clean Ratio	Q20	Q30
A_1	128,541,756	19,281,263,400	127,569,914	13,604,132,565	99.24%	98.36%	95.01%
A_2	89,333,144	13,399,971,600	88,641,850	11,625,646,313	99.23%	98.76%	95.87%
C_1	135,034,832	20,255,224,800	133,887,694	17,651,791,047	99.15%	98.63%	95.46%
C_2	77,318,490	11,597,773,500	77,040,194	9,515,338,581	99.64%	99.00%	96.47%

**Table 2:** Quality control for the RNA-seq data.

Sample	Raw Reads	Raw Bases	Clean Reads	Clean Bases	Clean Ratio	Q20	Q30
A_1	78,265,742	11,739,861,300	77,683,168	11,626,880,356	99.26%	98.25%	94.88%
A_2	53,208,116	7,981,217,400	52,829,996	7,906,568,770	99.29%	98.24%	94.80%
A_3	121,439,410	18,215,911,500	120,564,720	18,063,425,239	99.28%	98.23%	94.76%
C_1	102,222,314	15,333,347,100	101,316,102	15,149,604,968	99.11%	98.10%	94.56%
C_2	36,264,690	5,439,703,500	35,877,070	5,370,251,031	98.93%	98.19%	94.77%
C_3	101,895,462	15,284,319,300	101,061,120	15,133,341,000	99.18%	98.17%	94.70%

**Table 3:** Information on primers used in this study.

Primer	Sequence (5′–3′)	Product Size
*ANKRD2*-F	CCTGAGAGTCCGTCCTTAC	141 bp
*ANKRD2*-R	CCGTTTCTTCTGCTTGCGT	
*PLEKHA4*-F	GGCAATGCTCTCAGAAGGGA	118 bp
*PLEKHA4*-R	AATGGCCAGAGAGGACGAAC	
*ZNF503*-F	CGCTCTCTGGAAATAGCTCCG	122 bp
*ZNF503*-R	CTTGATGGGCAGGCGGTTAG	
*ANKRD1*-F	AGAAGAAAGGCAGTGGGGATG	121 bp
*ANKRD1*-R	ACAAAGTGGACCGGAAGTGT	
*BTG2*-F	GCATCCGCATCAACCACAAG	217 bp
*BTG2*-R	TTCTTGCAGGTGAGAAGCCC	
*FOS*-F	TACAGCCCACCCTAGTCTCC	71 bp
*FOS*-R	AGTAGGGACTCCATAGGGGTG	
*FOXO1*-F	ACCCCACAAGGTTTCCGATG	90 bp
*FOXO1*-R	AGTGTCCCCTCTCTTTCCAAC	
*LMOD3*-F	CCACCTTGTCCCCAGAAGAG	129 bp
*LMOD3*-R	GGTCGAAGTTCCCTGTTGGT	
*UCP3*-F	CGGACCACTCCAGCATCATT	186 bp
*UCP3*-R	CTTCCTCTCTGGCGATGGTC	
*GAPDH*-F	CCACGAGAAGTATAACAACACC	120 bp
*GAPDH*-R	GTCATAAGTCCCTCCACGAT	

## Data Availability

The data presented in the study are deposited in the NCBI repository, https://www.ncbi.nlm.nih.gov/sra/PRJNA1035797 (accessed on 7 November 2023) and the accession number: PRJNA1035797.

## Supplementary Materials

The following supporting information can be downloaded at: https://www.mdpi.com/article/10.3390/ijms25010158/s1.
