# Supplementary material for: Integration of ATAC-Seq and RNA-Seq Analysis to Identify Key Genes in the Longissimus Dorsi Muscle Development of the Tianzhu White Yak
Source: Int J Mol Sci. 2023 Dec 21;25(1):158. doi: 10.3390/ijms25010158 (PMC10779322; doi:10.3390/ijms25010158)
Supplement: Supplementary file 1 [file ijms-25-00158-s001.zip › Figure S1 Reads distribution.pdf]

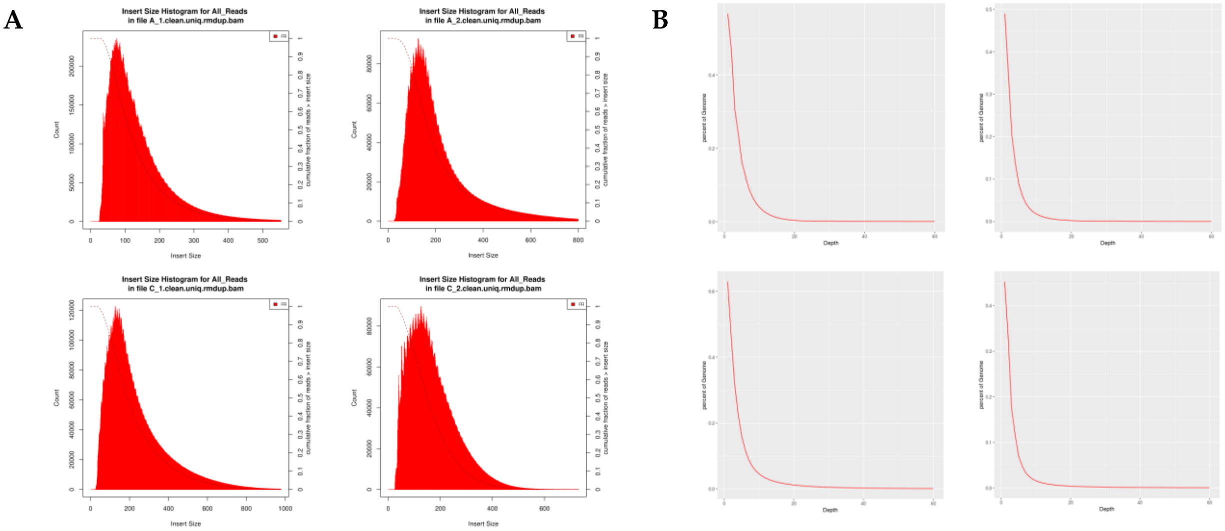

Figure S1. Fragment length distribution map. (**A**) Insert size histogram for each sample. (**B**) Fragment depth of each sample.
